# Effects of different volumes of a water-soluble and biodegradable nesting material on behavior, pain, glycemia, farrowing traits, and performance in crated sows

**DOI:** 10.1007/s11259-026-11451-6

**Published:** 2026-08-08

**Authors:** Matheus S. Monteiro, Bruno B. D. Muro, Marcos V. B. Nicolino, Ana L. B. Mezzina, Giovanni L. S. França, Rafaella F. Carnevale, Fernanda M. dos Santos, Ana C. R. Oliveira, Nádia A. C. Gomes, Caroline Veloso, Flávio A. de Coelho, José E. Martinez, Cecília A. F. Melo, Erich H. C. Inácio, Ana V. H. S. Escaler, Flávia S.S. Tavares, Gabriel T. Takita, Lidiane C. Costa, Thiago Bernardino, Cesar A. P. Garbossa

**Affiliations:** 1 Department of Animal Nutrition and Production, School of Veterinary Medicine and Animal Sciences, University of São Paulo (USP), Campus Pirassununga, Avenida Duque de Caxias Norte, nº 255, 13635-900 Pirassununga, SP Brazil; 2 Nerthus Pesquisa e Desenvolvimento LTDA, São Carlos, SP Brazil; 3 Graduate Program in Materials Science and Engineering, Federal University of Sao Carlos, São Carlos, SP Brazil; 4 Department of Veterinary and Biosciences, Faculty of Veterinary Medicine, Ghent University, Ghent, Belgium; 5 Department of Materials Engineering, Federal University of São Carlos, São Carlos, SP Brazil; 6 Center of Characterization and Development of Materials (CCDM), Federal University of SP, São Carlos, Brazil; 7 Centro Universitário Padre Anchieta, Jundiaí, SP Brazil

**Keywords:** Ethogram, Glycemia, Nest-building, Swine, Weaning weight

## Abstract

This study evaluated the effects of providing 3–15 kg of a soluble and biodegradable biopolymer-based enrichment material (blend of biodegradable polymers and natural fibers) to prepartum sows housed in farrowing crates. Fifteen sows were blocked by body weight and backfat thickness and assigned to three treatments: no enrichment material (CONT), 3 kg of enrichment material (BN3), or 15 kg of enrichment material with added nutritional value (BN15; 14.50 MJ ME/kg; composition: a blend of biodegradable natural polymers and natural fibers). The material was supplied 24 h before the expected farrowing date. Behavioral observations were performed during the 18 h preceding farrowing. Sow and piglet blood glucose concentrations were determined from venipuncture of auricular vein. Pain-related facial expressions, piglet birth intervals, and litter performance parameters were also evaluated. Behavioral analysis revealed significant differences between treated and control sows, with BN3 and BN15 sows showing reduced ventral lying, redirected nest-building behaviors, floor interaction, and postural changes during the pre-farrowing period (*p* < 0.05). A quadratic relationship was observed between piglet birth order and birth interval (*p* < 0.001), birth interval was longer early in parturition, decreased in the middle stages, and increased at the end of parturition. In addition, compared with CONT sows, BN15 sows provided exhibited shorter birth intervals (*p* = 0.030), while BN3 sows showed a tendency toward shorter birth intervals (*p* = 0.084). Sows in BN15 tended to have higher glycemia at farrowing onset than CONT (96.6 vs. 80.2 mg/dL; *p* = 0.022). Piglets from BN3 and BN15 tended to have higher birth glycemia than CONT (44.06 and 43.79 vs. 37.92 mg/dL; *p* < 0.10). Pain scores assessed through facial expressions/grimace score were lower (*p* < 0.10) in BN3 and BN15 than in CONT between 120 and 240 min after farrowing onset. Overall, providing biopolymer-based enrichment material, improved behavioral and physiological indicators associated with sow welfare.

## Introduction

The concepts of sustainability and animal welfare have increasingly shaped animal production systems through both policy and market demands (Mota-Rojas et al. 2023). Particular attention has been given to strategies that enhance the welfare and health of sows, as these animals not only remain in the herd for extended periods but also have a direct influence on the survivability, viability, and productive performance of their litters (Grimberg-Henrici et al. 2016).

From an animal welfare perspective, the peripartum period represents one of the most critical phases for both sows and piglets. This period is associated with the highest sow mortality rates (Monteiro et al. 2022) and with substantial piglet losses, largely due to complications during farrowing (Vanderhaeghe et al. 2011; Langendijk et al. 2019). Providing enrichment material to prepartal sows has been proposed as a management strategy to reduce stress, improve hormonal and behavioral patterns, and ultimately enhance sow health and productivity (Monteiro et al. 2025a, b).

Despite the domestication process, sows retain a strong intrinsic motivation to perform nest-building behaviors, including rooting, nosing, and pawing, in the period preceding farrowing (Jensen 1993; Yun et al. 2013, 2014; Plush et al. 2021). The opportunity to express these behaviors has been shown to positively influence farrowing and lactation performance (Yun and Valros 2015). Nest-building behavior is stimulated not only by endocrine changes but also by external factors, particularly the availability and quantity of enrichment material (Wischner et al. 2009). Accordingly, studies with pen-housed sows have demonstrated improved outcomes when larger amounts of enrichment material are provided (Yun et al. 2014).

Although the benefits of nesting materials are well established, their application in commercial pig production remains limited, especially in farrowing crates, where more than 90% of sows farrow. In these systems, commonly used materials such as straw or paper tend to fall through slatted floors, increasing the risk of slurry drainage blockage (Monteiro et al. 2025a, b; Plush et al. 2021; Rosvold et al. 2018). This risk is exacerbated when larger quantities of material are provided, which likely explains why most studies involving crated sows restrict material provision to approximately 2 kg (Monteiro et al. 2023). Alternative materials, such as burlap or jute sacks, are easier to manage and pose fewer risks to slurry systems; however, they are typically supplied in limited amounts and therefore fail to satisfy the sow’s strong motivation for nest-building before farrowing (Bolhuis et al. 2018; Monteiro et al. 2023). Moreover, confinement in farrowing crates is associated with elevated stress levels and increased expression of redirected nest-building behaviors (Yun and Valros 2015; Pan et al. 2022), which may influence behavioral and physiological responses to enrichment materials compared with those observed in pen-housed sows (Damm et al. 2003).

Although the provision of enrichment materials in conventional farrowing crates can be challenging because of housing design and manure handling systems, scientific evidence consistently supports the importance of enabling nest-building behavior, with reports of improved farrowing outcomes, colostrum intake, and litter performance when enrichment materials are provided (Yun and Valros 2015; Plush et al. 2021). Nest-building may also contribute to reduced peripartum pain, as oxytocin and prolactin are known to mediate maternal hypoalgesia (Mainau and Manteca 2011; Uvnäs-Moberg 2024). However, this relationship remains poorly understood, and the effects of enrichment material provision on sow pain have not yet been investigated.

In addition, nest-building behavior is energetically demanding, and sows frequently ingest portions of the provided materials, showing a preference for those with higher nutritional value (Edwards et al. 2019). Despite this, the impact of enrichment material provision on sow glycemia at farrowing onset, a biomarker of metabolic status and predictor of farrowing duration (Carnevale et al. 2023), has not yet been evaluated. This question is particularly relevant in modern hyperprolific sows, which often experience prolonged farrowing and increased energetic demands during parturition (Monteiro et al. 2025a, b). In this context, edible and energetic enrichment materials may provide an additional source of energy when consumed, potentially helping to maintain glycemia during the periparturient period and thereby supporting farrowing progression, piglet vitality, and sow health (Oliveira et al. 2020; Carnevale et al. 2023; Monteiro et al. 2025a, b).

Despite growing evidence supporting the benefits of nest-building opportunities, important knowledge gaps remain. Most studies involving crated-farrowing sows have evaluated only small quantities of enrichment material due to concerns regarding slurry system blockage, leaving the effects of larger quantities largely unexplored. Furthermore, although nesting behavior has been associated with improved welfare and farrowing outcomes, its potential influence on peripartum pain, particularly as assessed through facial expression scoring, has not been investigated. In addition, the effects of edible enrichment materials on sow glycemia status during farrowing remain unknown, despite the substantial energetic demands of nest-building activities and parturition. Therefore, this study evaluated the effects of providing different quantities of a novel edible, water-soluble enrichment material to crated-farrowing sows. We hypothesized that larger quantities of material would enhance nest-building behavior, improve farrowing and lactation performance, reduce indicators of peripartum pain, and improve glycemia at farrowing onset.

## Materials and methods

### Ethical note

The experimental procedure was approved by Ethics Committee for the use of Animals of the School of Veterinary Medicine and Animal Science of the University of São Paulo (CEUA / FMVZ-USP) under protocol number 9,652,211,024. Reduction of animals used enrolled in the trial was guaranteed by performing a sample size determination considering behavioral analysis. The sample size was determined based on previous studies evaluating nesting material provision in crated-farrowing sows. Behavioral responses reported by Monteiro et al. (2024) and Plush et al. (2021) demonstrated large treatment effects on nest-building activity, redirected nest-building/stereotypic behaviors, and postural changes during the prepartum period, with estimated Cohen’s d values (Eq. 1) ranging from approximately 1.4 to greater than 4.0. These findings were further supported by a systematic review and meta-analysis (Monteiro et al. 2023), which identified consistent and substantial behavioral responses to nesting material provision across multiple studies. Because this study evaluated a novel edible and water-soluble nesting material, it was considered exploratory in nature; therefore, a significance threshold of α = 0.10 was adopted to reduce the risk of Type II errors when assessing biologically relevant responses. Importantly, statistical trends were never interpreted in isolation but rather in the context of convergent evidence from behavioral, physiological, and productive outcomes. The sample size calculation was based on the primary biological question of the study, namely the comparison of each nesting material treatment (BN3 and BN15) with the conventional farrowing system without nesting material (CON), which represented the reference commercial condition. Accordingly, planned contrasts were performed to compare BN3 versus CON and BN15 versus CON.

Considering both the magnitude and consistency of these effects and the exploratory nature of the study, five sows per treatment were considered sufficient to detect biologically meaningful differences among treatments.

Equation (1):

$$\:d=\frac{\mathrm{Mean}_{\mathrm{CONT}}-\mathrm{Mean}_{\mathrm{Nest}}}{\sqrt{\frac{\mathrm{SD}_{\mathrm{CONT}}^{2}+\mathrm{SD}_{\mathrm{Nest}}^{2}}{2}}}$$

Where:

d = Cohen’s effect size; 

SD = Standard deviation;

CONT (sows without access to nest-building) and Nest (sows with access to nest-building).

Refinement was achieved by reducing social isolation to a minimum. All animals had visual and acoustic contact with other individuals throughout the whole experimental period. All sows had at least 12 h of contact with day light. This trial aims to develop an enrichment material for farmers to be in accordance with the Brazilian IN 113 and the European council directive 2008, which dictate the mandatory provision of an enrichment material, but come up against structural issues such as blocked pipes which hinders the use of natural materials such as straw and hay.

### Animals, management, housing and feeding

The study was performed at an experimental unit, located in Pirassununga, São Paulo, Brazil. A total of 15 cross-bred parity 4 sows (DanBred - DB 90^®^, Patos de Minas, MG, Brazil) and their litters were included in the trial. During gestation sows were kept in crates (2.10 m of length x 0.65 m of width x 1.00 m of height), with natural ventilation and light and partly slated floor. All sows were fed twice a day (07am and 14pm) 1.35 kg (2.7 kg per day) of a standard gestation diet (12.54 MJ ME/kg, 14.49% CP, 3.58% CF, 3.45% fat, 0.6% Dig Lys). The same gestation diet was provided from the entry in the farrowing room until farrowing. Throughout the whole experimental period females had *ad libitum* access to water in nipple drinkers. Approximately one week before the expected farrowing date, sows were transferred to the farrowing unit and housed individually in crates (sow area = 2.20 m of length x 0.55 m of width x 1.00 m of height, pen size = 2.40 × 1.85 m) which contained partially slatted metal flooring, a feeder trough, nipple drinker, and a closed creep area for piglets equipped with heating lamps. Sows had visual and acoustic contact with at least 6 other sows, that were housed in the same room. The farrowing unit had natural ventilation and light. Room temperature was controlled with plastic sidewall blinds. Following farrowing, sows were hand-fed 5 kg/day of a standard lactation diet (14.22 MJ ME/kg, 18.55% CP, 2.71% crude fiber, 6.48% fat, and 1.12% digestible lysine), with feed allowance subsequently adjusted according to appetite to ensure *ad libitum* feed. Feeders were checked approximately eight times daily, and additional feed was provided whenever feeders were empty to ensure unrestricted access to feed.

### Enrichment material

**Fig. 1:**
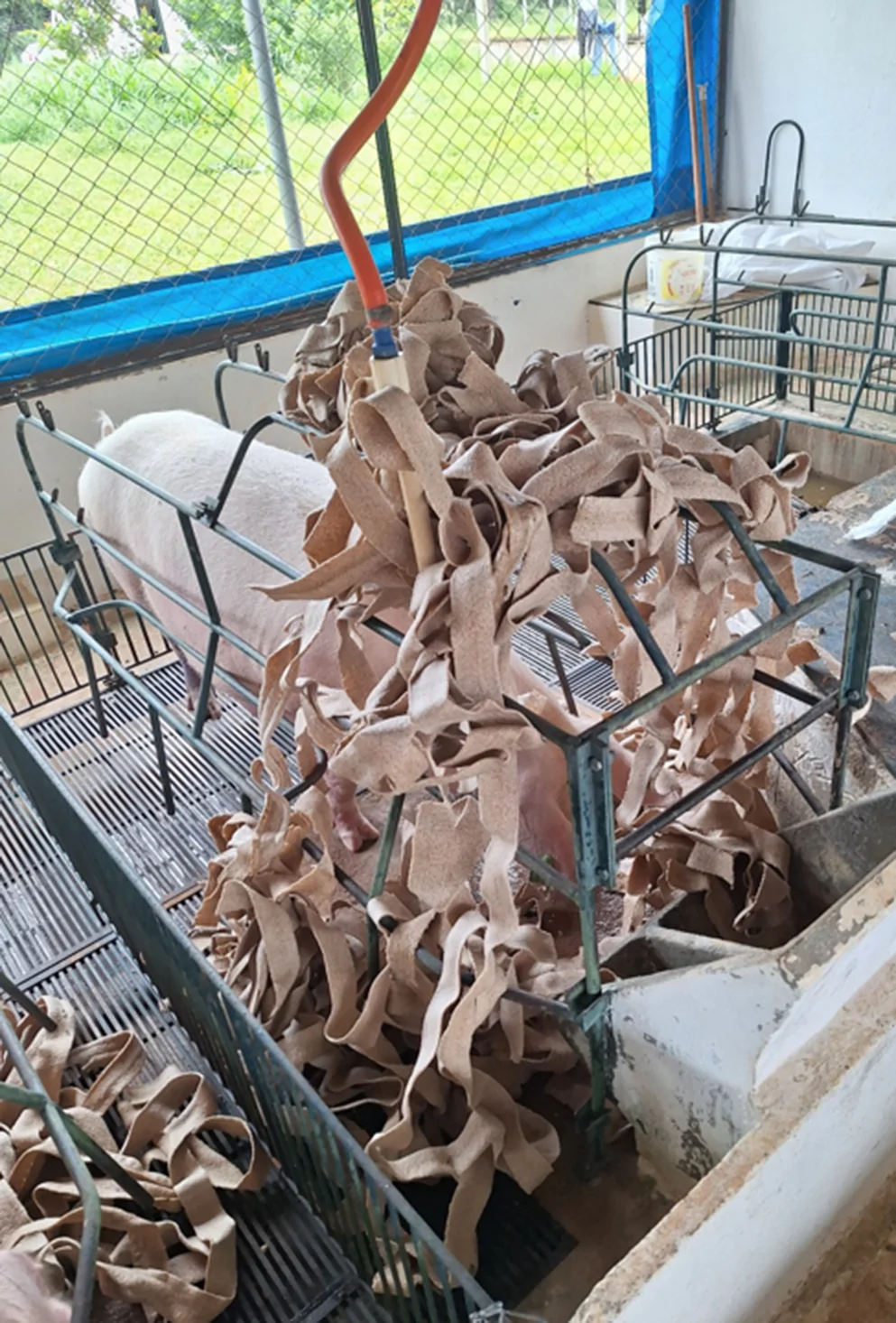
– Provision of 15 kg of biopolymer-based enrichment material (BioNest™, Nerthus, São Carlos, SP, Brazil − 14.50 MJ ME/kg, composition: blend of biodegradable polymers and natural fibers)

For the experiment a biopolymer-based enrichment material was used (BioNest™, Nerthus, São Carlos, SP, Brazil − 14.50 MJ ME/kg, composition: blend of biodegradable and natural polymers and natural fibers) to mimic the properties of natural enrichment materials (Fig. 1) without effluent pipe blockage constraints. This biopolymer-based material is water-soluble and fully biodegradable.

### Experimental design

At transference to the farrowing house sows were blocked according to weight and backfat thickness and allocated to one of the three treatments: CONT (*n* = 5): sows that had no access to any enrichment material or environmental enrichment; BN3 (*n* = 5): sows that were supplied with 3 kg of a biopolymer-based enrichment-material 24 h before the expected date of farrowing; BN15 (*n* = 5) sows that were supplied with 15 kg of a biopolymer-based enrichment-material 24 h before the expected date of farrowing. The enrichment material was provided on the floor in front of the sow feeder trough and also hung in the crate (Fig. 1). Expected farrowing date was estimated from the insemination closest to the final third of estrus, assuming a gestation length of 114 days. This procedure was adopted to minimize variation in the interval between material provision and the onset of parturition. The assigned quantity of enrichment material was provided once daily. If a sow did not farrow on the expected day, any remaining material was removed and replaced with a fresh quantity corresponding to the experimental treatment on the following day. This procedure was repeated until the onset of farrowing, ensuring continuous access to nesting material while maintaining a standardized quantity among sows. All females were closely monitored during the farrowing, with no use of manual obstetrical assistance (birth canal palpation) or oxytocin administration.

### Sow body composition and feed intake

To measure sow body composition during lactation, the backfat thickness and loin depth of were evaluated at transference to the farrowing crate and at weaning using an ultrasound scanner (3.5 MHz linear array probe; KX 5600 – Vet Chinson; China) at P2 position (∼6 cm on both sides of mid-line at the level of the last rib). Sows were also weighed at the same timepoints. All the feed provided to sow was weighed to calculate the feed intake. Feed refusals were collected and weighed daily, allowing individual feed intake to be calculated for each sow throughout lactation.

### Behavioral observations and pain assessment scale based on facial expressions

For behavior observations, all sows were recorded from the supply of the enrichment material until the onset of the farrowing. To standardize the analysis behaviors were analyzed only in the 18 h before the farrowing onset (first piglet born). Cameras Mibo IC3 (Intelbras company, São José, Santa Catarina, Brazil) in Full HD (1920 × 1080 pixels) resolution and at 30 frames per second were used to record the videos. Each sow was recorded by a camera, and digital cameras were mounted on the rear walls of the farrowing room at an approximate height of 2.5 m above the floor. Cameras were positioned to provide an oblique viewing angle of approximately 45° relative to the sow, allowing continuous observation of postural changes, behavioral activities, and interactions with the enrichment material while minimizing visual obstruction from the farrowing crate structure. During analysis, behaviors were adapted from Rosvold et al. 2018; Monteiro et al. 2025a, b and classified in accordance with the ethogram in Table 1 and noted with Boris software (Friard and Gamba 2016). Postural behaviors (standing, sitting, ventral lying, and lateral lying) were recorded as mutually exclusive state events, such that only one posture could be assigned at any given time. Nest-building and redirected nest-building behaviors were also coded as mutually exclusive state events, allowing calculation of the proportion of observation time spent performing each behavior. To quantify behavioral frequency, bar biting and postural changes were additionally recorded as point events, enabling determination of the number of occurrences during the observation period. Behavioral data were subsequently exported from BORIS for statistical analysis. A behavior was included in the analysis only if it was performed for at least 10 s. Repeated occurrences of the same behavior separated by less than 30 s were considered a single event. Behavioral data were expressed as the percentage of total observation time (18 h) that sows spent performing each behavior. In addition, behavior was analyzed across successive time intervals relative to the onset of farrowing (defined as time 0): (i) − 18 to − 12 h, (ii) − 12 to − 6 h, (iii) − 6 to − 2 h, and (iv) − 2 to 0 h before farrowing.

**Table 1:** Ethogram used for behavioral analysis of sows during the pre-farrowing period. Behaviors were identified from video recordings and coded using BORIS software. Postural behaviors, nest-building activities, redirected nest-building behaviors, and feeding- and drinking-related activities were classified according to the definitions presented below

Behavior	Description
Postural behaviors	
Standing	Upright posture with weight supported by all four legs
Lateral lying	Lie on the right or left side with the udder exposed
Ventral lying	Lie on udder
Sitting	Rump on the ground with front legs straight
Postural changes	Any movement that changes between the postures listed above and from lateral Lying right to left and vice-versa
Nest-building behavior	Back-and-forth movements with the nose, front legs to the material or on the ground. Chew the material
Engaged with material	Nest-building behavior directed to the material
Interaction with the floor	Nest-building behavior directed to the floor
Redirected nest-building behaviour: Bar biting and cage interaction	Biting bars and surrounding structures (e.g. feeder) with mouth
Food-related	Eating food in feed bin. Not including BioNest
Drinking	Water consumption

The facial expression of pain were also assessed. In order to do it, pictures of the sow’s face were taken every 30 min using a smartphone from onset of the farrowing until 300 min after farrowing onset. Five parameters were analyzed as described by Navarro et al. (2020) tension above eyes, snout angle, neck tension, temporal tension and ear position, cheek tension. Each of the parameters were scored as 0, 1 or 2, with 2 being the worst degree of pain. Each photograph was taken from approximately 1 m of distance and allowed visualization of five predefined parameters (tension above eyes, snout angle, neck tension, temporal tension, ear position, cheek tension). Image acquisition was standardized according to the examples described by Navarro et al. (2020). Photographs were obtained only when the sow’s face was fully visible, allowing assessment of all facial action units. If the sow’s posture prevented full visualization of the face, the observer repositioned to obtain an alternative angle and captured the image. The pictures were analyzed by 3 blinded independent evaluators. Inter-observer reliability among three raters was substantial: mean pairwise Pearson correlation = 0.71, mean quadratic weighted kappa = 0.64, indicating acceptable agreement for pain assessment based on facial expressions.

### Farrowing traits and piglet performance

Farrowing duration was considered as the interval of time elapsed between the birth of the first and last piglet. Birth interval was considered as the time elapsed between the birth of two consecutive piglets. At birth, piglets were classified as liveborn, stillborn, or mummified. Piglet mortality was calculated for the first 24 h and whole lactation period (21 days). At birth, all piglets were individually identified with ear tags, weighed, dried using a commercial drying powder, and reweighed 24 h later to estimate colostrum intake using the equation described by Theil et al. (2014). Sows’ colostrum yield was also estimated by summing the individual piglets’ colostrum intake. Piglets were also weighed at weaning 21 days.

### Glycemia and temperature measurements

The piglets’ and sows’ glycemia were assessed using a portable glucometer (Accu-Chek Guide MeterTM, Roche Diabetes Care, Inc) as described by Carnevale et al. (2023). The venous blood samples were collected by puncture of the auricular vein with a needle in both piglet and sows. Piglets’ glycemia was analyzed at birth and 24 h after birth, at the moment of the second glycemia measurement piglets’ rectal temperature was also measured with a digital thermometer. Sows’ glycemia was analyzed at the onset of farrowing and 300 min after the onset of farrowing.

### Statistical analyses

The statistical analyses were conducted using R software (version 4.2.1; R Core Team, Vienna, Austria). All models’ residuals were assessed for normality and homoscedasticity by visual inspection of histograms and Q-Q plots and with Shapiro Wilk and Barlett tests, respectively.

Sow-level outcomes, including sow glycemia, farrowing duration, body weight, backfat thickness, loin depth, feed intake, and colostrum yield, total born and born alive were analyzed using analysis of variance models. Farrowing duration and colostrum yield models included total born piglets as a covariate. Birth weight, colostrum intake, piglets glycemia, rectal temperature, weaning weight, and pain assessment scale based on facial expression were analyzed using linear mixed-effects models fitted with the lmer function. For piglet-level outcomes, sow was included as a random effect to account for the hierarchical structure of the data. For pain assessment scale based on facial expressions both sow and observer included as random effects to account for repeated observations within sows and potential variability among observers.

Piglet birth intervals were analyzed using linear mixed-effects models including orthogonal polynomial contrasts for birth order (linear and quadratic terms), treatment, and their interactions. Sow was included as a random effect to account for repeated measurements within litters. Stillbirth rate, mummified fetuses, and piglet mortality were analyzed using generalized linear mixed-effects models fitted with the glmer function assuming a binomial distribution. Statistical significance was declared at *P* ≤ 0.05, whereas values between 0.05 and 0.10 were considered trends.

Principal component analysis (PCA) was performed to reduce the dimensionality of sow behavioral data, transforming multiple correlated variables into a set of linearly uncorrelated principal components. Prior to analysis, the data were standardized (mean- centered and scaled to unit variance) to ensure equal contribution of all variables to the principal components. The PCA biplot visualized both variable loadings and individual sow scores, with experimental groups differentiated by color. 95% confidence ellipses were generated for each treatment group to assess group separation in multivariate space, while treatment centroids represented the mean position of each group in the principal component space. To maintain consistency with the standardized multivariate dataset, treatment effects on the behavioral profile were assessed by PERMANOVA using Euclidean distances calculated from the centered and scaled behavioral variables. The significance of treatment effects was assessed using 999 permutations. Specifically, the following a priori contrasts were evaluated: i-) CONT vs. BN3; ii-) CONT vs. BN15. These contrasts were defined before data analysis based on the study objective of quantifying the effects of providing different quantities of enrichment material relative to conventional farrowing management without enrichment material. Data are presented as LSMEANS with the largest standard error. The difference between the mean values was considered statistically significant when *P* < 0.05 and trend when *p* > 0.05 and < 0.10.

## Results

### Body condition and feed intake

The experimental groups have similar (*p* > 0.10) weight, backfat thickness and loin depth after moving to farrowing room. During lactation, the experimental groups did not impact (*p* > 0.10) feeding intake, body weight, backfat thickness, or loin depth at weaning (Table 2).

**Table 2:** Sow bod weight, bod composition and lactation feed intake according to treatment

Variable	CONT	BN3	BN15	SEM	*p*–value: Contrast	
					CONT vs. BN3	CONT vs. BN15
Sow body weight at transference^1^ (kg)	278	280	280	12.5	0.991	0.927
Backfat thickness at transference^1^ (kg)	16.0	17.1	16.0	1.86	0.705	0.882
Loin Depth at transference^1^ (kg)	60.8	59.7	59.6	2.13	0.825	0.823
Body weight at weaning^2^ (kg)	269	265	259	12.30	0.822	0.561
Backfat thickness at weaning^2^ (kg)	16.0	15.4	15.5	1.77	0.988	0.796
Loin Depth at weaning^2^ (kg)	60.5	60.6	60.5	2.74	0.984	0.852
Lactation feed intake^3^ (kg)	6.67	6.47	6.45	0.49	0.760	0.726

^1^Transference: Day of moving the sows from gestation to the farrowing room (7 days before farrowing). These measurements obtained at transfer to the farrowing crate were collected before nesting material provision and were used to verify the comparability of treatment groups at the beginning of the experimental period. ^2^Measurements obtained at weaning reflect the effects of treatment throughout lactation. ^3^Lactation feed intake represents the average daily feed intake during the 21-day lactation period. CONT: sows that had no access to any nesting material; BN3: sows that were supplied with 3 kg of a biopolymer-based nesting-material; BN15: sows that were supplied with 15 kg of a biopolymer-based nesting-material. Values are presented as least-squares means with their respective standard errors of the mean (SEM)

### Behavioral observations

**Fig. 2:**
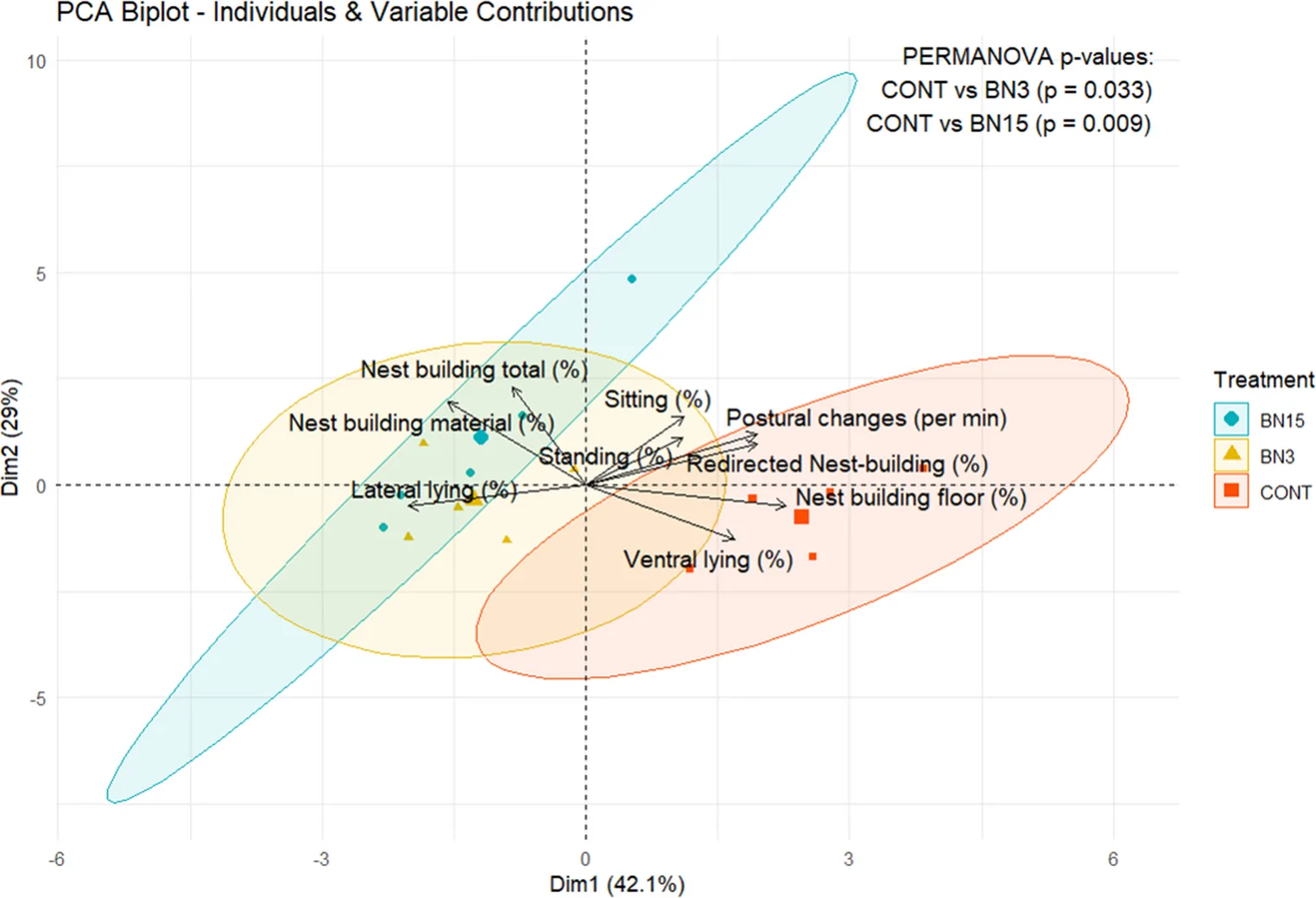
Biplot graph of Principal Component Analysis (PCA) sows pre-farrowing behavior. Variables are indicated as arrow and individuals are represented by group symbol. Ellipses represent 95% confidence regions for each treatment group’s multivariate distribution in principal component space and the symbol at the center of each ellipse represents the multivariate centroid (geometric center) of that treatment group in the principal component space. CONT: sows that had no access to any nesting material; BN3: sows that were supplied with 3 kg of a biopolymer-based nesting-material; BN15: sows that were supplied with 15 kg of a biopolymer-based nesting-material

The PCA biplot showed the distribution of treatments and behavioral variables along Dimension 1 (Dim1) and Dimension 2 (Dim2), corresponding to the first and second principal components, respectively. The first two dimensions of the principal component analysis (PCA) explained 71.1% of the total variation, with Dim1 accounting for 42.1% and Dim2 for 29%. The PCA revealed clear patterns in the relationship between behaviors and treatments – Fig. 2. Sows from the BN15 group were mainly distributed on the negative side of Dim1 and Dim2, which was associated with higher frequencies of nest-building behaviors. In contrast, the CONT was positioned predominantly on the positive side of Dim1, strongly associated with higher frequencies of redirected nest-building, increased postural changes, greater ventral lying, and more nest-building attempts focused on the floor. The BN3 group was located between the BN15 and CONT groups but showed a tendency to cluster closer to BN15, suggesting an intermediate behavioral profile. The variable lateral lying was positioned opposite to stress-related behaviors such as postural changes, redirected nest-building, and ventral lying. The PERMANOVA revealed significant differences (*p* < 0.05) in behavior in the BN3 and BN15 when compared to CONT sows.

**Fig. 3:**
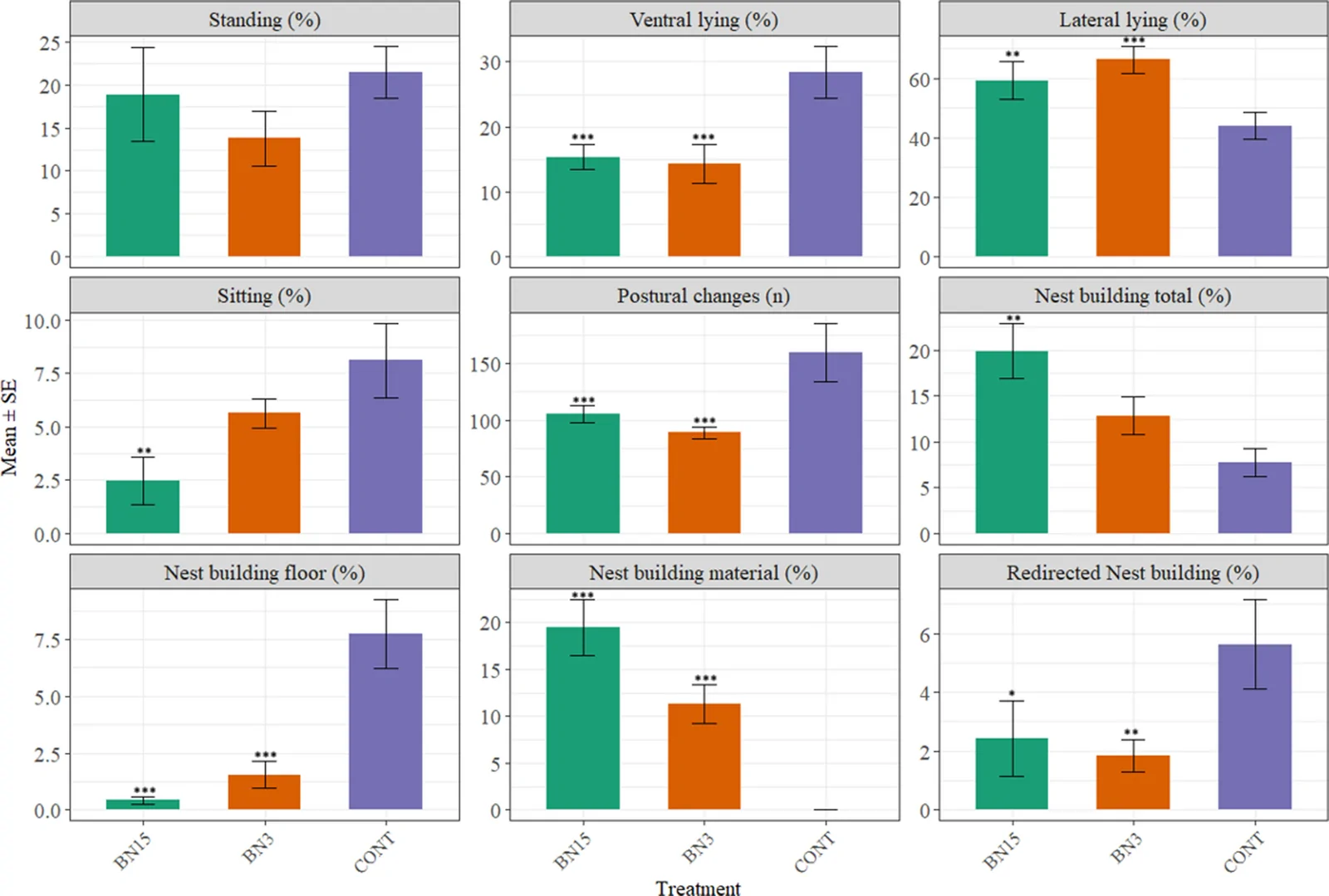
Behavior of sows in the 18 h before farrowing. Asterisks above BN3 or BN15 indicates statistical difference of the respective compared to CON group in contrast analysis. * *p* < 0.10 and > 0.05; ** *p* < 0.05 and > 0.01; *** *p* < 0.01. CONT: sows that had no access to any nesting material; BN3: sows that were supplied with 3 kg of a biopolymer-based nesting-material; BN15: sows that were supplied with 15 kg of a biopolymer-based nesting-material

The results regarding mean time sows spent performing a behavior in the 18 h before farrowing onset are depicted in Fig. 3. The experimental groups did not impact (*p* > 0.10) the time spent in standing position. For the other postural behaviors, sows in CONT group spent more time in ventral lying (*p* < 0.05) and less time in lateral lying (*p* < 0.05) when compared to both BN3 and BN15. Sows in the BN15 spent less time (*p* = 0.013) sitting than CONT sows. Providing nest-building material reduced (*p* < 0.05) postural changes before farrowing, irrespective of the group (BN3 and BN15) when compared to CONT dams. BN15 sows spent more time expressing nest-building behavior (*p* = 0.003) when compared to CONT, while access to nest-building material reduced (*p* < 0.05) the time spent in floor nest-building, in both BN3 and BN15 sows compared to CONT. The BN3 reduced (*p* = 0.045) redirected nest-building and BN15 tended to reduce the time spent performing redirected nest-building (*p* = 0.083) when compared to CONT.

**Fig. 4:**
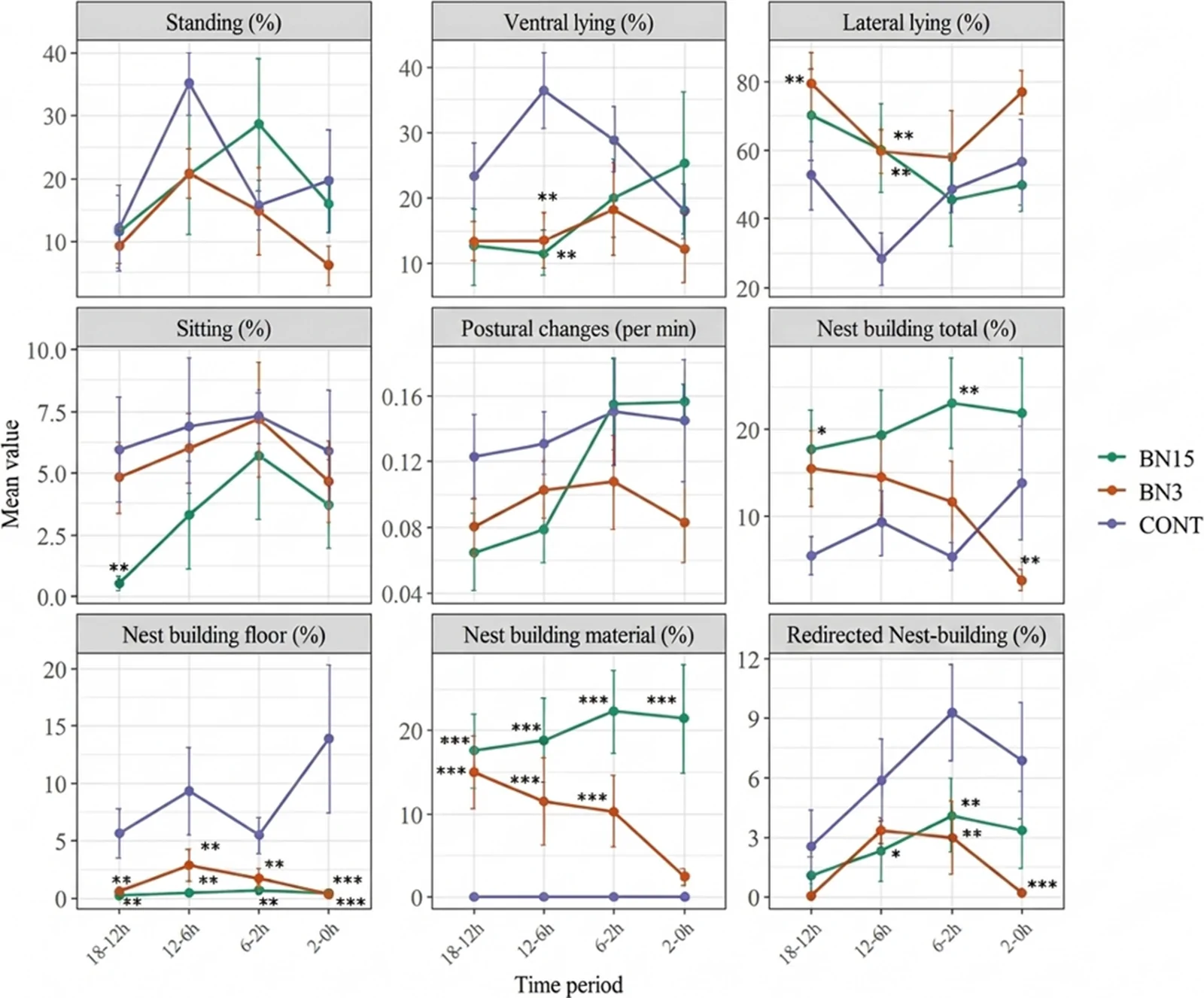
Behavior of sows in different periods before farrowing. Asterisks above BN3 or BN15 indicates statistical difference of the respective compared to CONT group in contrast analysis. * *p* < 0.10 and > 0.05; ** *p* < 0.05 and > 0.01; *** *p* < 0.01. CONT: sows that had no access to any nesting material; BN3: sows that were supplied with 3 kg of a biopolymer-based nesting-material; BN15: sows that were supplied with 15 kg of a biopolymer-based nesting-material

The pre-farrowing behavior segregated by periods can be visualized in Fig. 4. For the period of 12–6 h before farrowing, BN3 and BN15 had a reduced (*p* < 0.05) time spent in ventral lying and a higher (*p* < 0.05) time spent in lateral lying. BN15 sows also reduced (*p* = 0.039) the time spent sitting in the period of 18–12 h before farrowing when compared to CONT sows. Sows in the BN3 and BN15 group spent less time performing redirected nest-building in the periods of 6–2 h and 2–0 h before farrowing.

### Pain assessment scale based on facial expressions

**Fig. 5:**
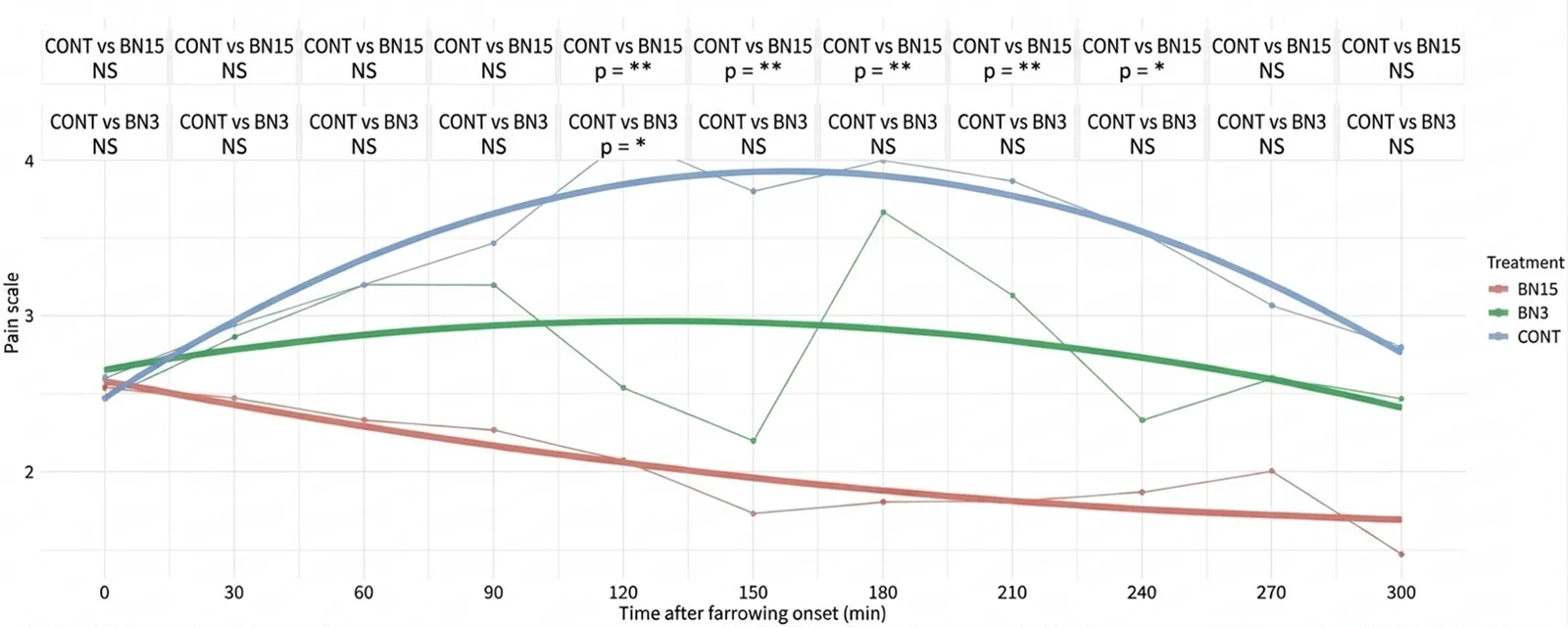
Pain score over time after farrowing onset. Pain was assessed using a facial expression scale based on five facial action units (i-tension above the eyes, ii-snout angle, iii-neck tension, iv-temporal tension and ear position, and v- cheek tension), each scored from 0 (no pain) to 2 (highest pain expression). The y-axis represents the sum of the scores for the six facial action units evaluated. Higher scores indicate greater pain expression. CONT: sows that had no access to any nesting material; BN3: sows that were supplied with 3 kg of a biopolymer-based nesting-material; BN15: sows that were supplied with 15 kg of a biopolymer-based nesting-material. * *p* < 0.10 and > 0.05; ** *p* < 0.05 and > 0.01; *** *p* < 0.01. CONT: sows that had no access to any nesting material; BN3: sows that were supplied with 3 kg of a biopolymer-based nesting-material; BN15: sows that were supplied with 15 kg of a biopolymer-based nesting-material

The impacts of providing 3–15 kg of enrichment material on sows’ pain assessment scale based on facial expression scale can be visualized in Fig. 5. At farrowing onset, sows from the different experimental groups had similar (*p* > 0.10) facial expressions of pain scale patterns. After 120 min, a reduction in the facial expression score was observed in the BN15 group (*p* = 0.034), while the BN3 group showed a tendency toward a reduction in facial expression score (*p* = 0.100) when compared to CONT. The BN15 group exhibited lower facial expression scores (*p* < 0.05) from 120 to 210 min after the onset of farrowing and tended (*p* = 0.099) to have lower facial expression scores at 240 min after the onset of farrowing compared with the CONT group.

### Farrowing traits, piglet colostrum intake, and sow colostrum yield

**Fig. 6:**
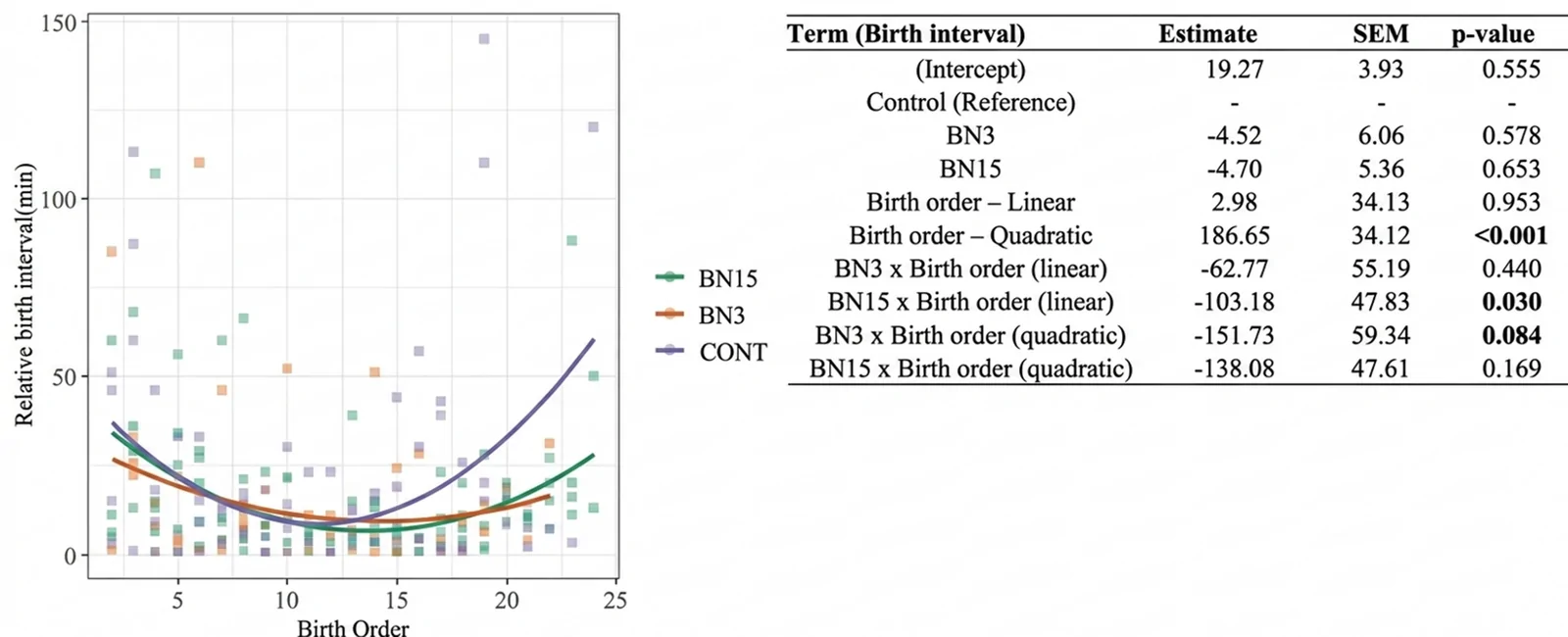
Relative birth interval across the different birth orders. CONT: sows that had no access to any nesting material; BN3: sows that were supplied with 3 kg of a biopolymer-based nesting-material; BN15: sows that were supplied with 15 kg of a biopolymer-based nesting-material

Farrowing traits and sows and piglets’ glycemia patterns are depicted in Table 3. Sows in the BN15 group had higher glycemia at farrowing onset (CONT vs. BN15, *p* = 0.022), while BN3 did not impact this variable (CONT vs. BN15, *p* = 0.403). At 300 min after farrowing onset, no differences in glycemia were observed among treatments (*p* > 0.10). The farrowing duration was not affected by the experimental groups. The piglets’ birth interval along the different birth orders can be visualized in Fig. 6. The estimated intercept of the regression model for the CONT group was 19.27 min. A significant quadratic relationship was observed between birth order and birth interval (*p* < 0.001), indicating that birth intervals were longer at the beginning of farrowing, decreased during the middle stages of parturition, and increased again toward the end of farrowing. Compared with the CONT group, sows in the BN15 group showed a significant linear reduction in birth interval across successive birth orders (*p* = 0.030), indicating shorter intervals between piglets as farrowing progressed. In addition, the interaction between BN3 and the quadratic term of birth order tended to be significant (*p* = 0.084), suggesting a modification of the curvilinear pattern of birth intervals, in order to reduce the birth interval, throughout parturition compared with the CONT group.

**Table 3:** Farrowing traits, sow glycemia, piglet glycemia and rectal temperature, and lactational performance of sows

Variable​s	CONT​	BN3​	BN15​	SEM​	p –value: Contrast​	
					CONT vs BN3​	CONT vs BN15​
Farrowing traits						
Farrowing duration (min)	378.4	254.2	351	84.0	0.295	0.788
Piglets born per litter (n)	20.8	18.7	20.6	2.79	0.498	0.654
Piglets born alive per litter (n)	17.5	17.3	18.4	2.62	0.963	0.774
Stillborn per litter (%)	9.4	3.8	4.64	3.22	0.165	0.246
Mummified per litter (%)	4.9	7.76	4.94	2.70	0.509	0.437
Birth weight (kg)	1.18	1.23	1.21	0.096	0.716	0.770
Sows glycemia						
Sows initial glycemia (mg/dL)	80.2	85.6	96.6	4.41	0.403	0.022
Sows glycemia - 300 min after farrowing onset (mg/dL)	90.2	85.8	100.4	7.02	0.665	0.324
Piglets glycemia and temperature						
Piglets’ birth glycemia (mg/dL)	37.92	43.79	44.06	2.233	0.099	0.085
Piglets’ blood glycemia after 24h of birth (mg/dl)	85.5	87.0	76.5	11.61	0.926	0.524
Piglets’ temperature after 24h of birth (°C)	38.3	38.5	38.2	0.30	0.675	0.845
Performance						
Colostrum intake (g)	284	287	290	16.5	0.895	0.776
Colostrum yield (g)	4721	4817	4890	496	0.894	0.804
Piglets’ temperature after 24h of birth (°C)	38.3	38.5	38.2	0.30	0.675	0.845
Piglets weaned per litter (n)	12.0	12.7	13.6	1.08	0.795	0.484
Piglets weaning weight (kg)	5.41	6.40	5.71	0.348	0.067	0.550
Litter weaning weight (kg)	67.7	78.9	73.9	12.10	0.506	0.673
Pre-weaning mortality (%)	33.8	27.3	26.8	7.58	0.535	0.437

CONT: sows that had no access to any nesting material; BN3: sows that were supplied with 3 kg of a biopolymer-based nesting-material; BN15: sows that were supplied with 15 kg of a biopolymer-based nesting-material. Values are presented as least-squares means with their respective standard errors of the mean (SEM)

There were no differences among the experimental groups (*p* > 0.10) in total piglets born, born alive, stillbirth, mummified, and birth weight – Table 4. Piglets born from BN3 and BN15 sows tended to have higher blood glucose concentrations at birth compared with piglets born from CON sows (CON vs. BN3, *p* = 0.099; CON vs. BN15, *p* = 0.085). However, these differences were no longer evident at 24 h after birth, when neither glycemia nor rectal temperature differed among treatments (*p* > 0.10). The experimental groups did not differ (*p* > 0.10) for colostrum intake, colostrum yield and mortality. The BN3 tended (*p* = 0.067) to an increased weaning weight when compared to CONT. Despite this, litter weight was similar (*p* > 0.10) across the experimental groups.

## Discussion

This study investigated whether providing increased quantities of a novel biopolymer-based enrichment material could enhance nest-building behavior and welfare-related outcomes of crated sows during the peripartum period. We hypothesized that supplying a larger quantity of enrichment material would increase nest-building activity, improve farrowing and lactational outcomes, reduce pain-related facial expression scores, improve the glycemia of sows and piglets, and ultimately contribute to improved farrowing traits. The results partially supported this hypothesis. Providing enrichment material increased nest-building behavior, reduced redirected nest-building behavior and postural changes, increased sow glycemia at farrowing onset and piglet glycemia at birth, reduced facial expression scores associated with pain, and increased piglet weaning weight. Several of these responses were more pronounced in sows provided with the larger quantity of material (BN15), which exhibited higher glycemia at farrowing onset, lower facial expression scores, and a more favorable pattern of birth intervals throughout farrowing compared with control sows. However, despite these behavioral and physiological changes, enrichment material provision did not affect overall farrowing duration, colostrum yield, feed intake, or piglets mortality.

The changes observed in redirected nest-building behaviors indicate that access to enrichment material altered the behavioral repertoire of sows during the pre-farrowing period. The multivariate analysis supported these findings by showing a clear separation between control sows and those provided with enrichment material. The PCA indicated that control sows were more closely associated with redirected nest-building behaviors, nest-building directed toward the floor, and increased postural activity, whereas BN3 and BN15 sows were associated with nest-building directed toward the provided material and increased lateral lying. These results suggest that enrichment material modified the overall behavioral profile of prepartum sows, supporting the interpretation that it facilitated the expression of species-specific behaviors.

In line with previous reports showing that enrichment materials reduce redirected nest-building behaviors and promote nest-building activity (Edwards et al. 2019; Plush et al. 2021; Rosvold et al. 2018), sows without enrichment material in the present study showed a progressive increase in redirected behaviors as farrowing approached. Redirected nest-building directed toward the floor was most pronounced during the final hours before parturition, whereas sows provided with 3–15 kg of biopolymer-based enrichment material exhibited lower levels of these behaviors and greater engagement with the enrichment substrate. These findings suggest that the provision of enrichment material modifies prepartum behavioral expression and allows sows to direct nest-building-related activities toward the available substrate.

Although the present study did not directly assess emotional state or physiological indicators of stress, the observed behavioral patterns are consistent with previous reports linking enrichment materials provision to improved welfare-related outcomes in farrowing sows (Edwards et al. 2019; Plush et al. 2021; Rosvold et al. 2018; Monteiro et al. 2023, 2025a, b). In line with previous studies using conventional nesting materials, the provision of enrichment material in the present study altered prepartum behavioral expression. Edwards et al. (2019), providing approximately 7 kg of lucerne hay, Rosvold et al. (2018), using 2 kg of long-stem barley straw, and Plush et al. (2021), using 2 kg of chopped straw, all reported increases in nest-building behavior accompanied by reductions in redirected nest-building behaviors. Similarly, the systematic review of Monteiro et al. (2023), which compiled studies evaluating a wide range of enrichment materials, concluded that nesting material provision consistently stimulates nest-building activity and reduces redirected behaviors. Consistent with these findings, sows provided with 3–15 kg of the biopolymer-based material in the present study exhibited lower levels of redirected nest-building behavior and greater engagement with the nesting substrate. These results suggest that the biodegradable and water-soluble material promoted behavioral responses comparable to those reported for conventional materials such as straw and hay, while potentially overcoming practical limitations associated with the use of natural materials in intensive farrowing systems, particularly the risk of slurry system blockage.

Providing enrichment material, irrespective of quantity, altered sow postural behavior during the pre-farrowing period, with sows spending less time lying ventrally, more time lying laterally, and exhibiting fewer postural changes. In contrast, control sows spent more time lying ventrally and in a seated posture. These postural patterns have previously been associated with increased restlessness and discomfort during the prepartum period (McGlone and Morrow-Tesch 1990; Melišová et al. 2014). Therefore, the reduction in postural changes observed in enriched sows may indicate improved comfort during the hours preceding farrowing. This interpretation is supported by the lower facial expression scores indicative of pain observed in BN15 sows, suggesting that providing a larger quantity of enrichment material was associated with changes in both behavioral and welfare-related indicators around parturition.

The broader literature suggests that these behavioral changes may have implications beyond sow welfare. A recent meta-analysis reported that the provision of nesting material is generally associated with reduced piglet mortality and improved maternal care, while also increasing piglet weaning weight (Monteiro et al. 2023). However, these benefits are not consistently observed across all studies and likely depend on the type and quantity of material provided as well as housing and management conditions. In the present study, enrichment material did not affect pre-weaning mortality, colostrum intake, or colostrum yield. Nevertheless, piglets born from sows provided with 3 kg of nesting material were heavier at weaning, suggesting that some of the benefits associated with improved maternal welfare and behavioral expression may be reflected in offspring growth performance even when survival-related outcomes remain unchanged.

Another distinguishing feature of the tested enrichment material is that it contains ingredients with nutritional value, including starch-based components, and the tested material have a calculated metabolizable energy content of 14.50 MJ/kg, exceeding that of the gestation diet. Previous studies have shown that edible and nutritionally valuable nesting materials are preferred by prepartum sows compared with non-nutritive substrates (Edwards et al. 2019). Because both nest-building behavior and parturition are energetically demanding processes (Vallet et al. 2013; Feyera and Theil 2017), the nutritional characteristics of the enrichment material may be relevant when interpreting the physiological responses observed in the our study. Indeed, previous research has demonstrated that increasing energy availability around farrowing can reduce farrowing duration and piglets birth interval (Oliveira et al. 2020; Carnevale et al. 2024).

In the present study, sows provided with the larger quantity of enrichment material exhibited higher glycemia at the onset of farrowing and a more favorable pattern of birth intervals, particularly among later-born piglets. Although overall farrowing duration was not affected, the shorter birth intervals observed as farrowing progressed suggest that the effects of enrichment material were expressed in the dynamics of piglet expulsion rather than in the total duration of parturition. Given that prolonged cumulative birth intervals have been associated with poorer piglet outcomes and reduced survivability (Schoos et al. 2023), these changes may be biologically relevant.

The mechanisms underlying these responses were not directly evaluated. However, they may be related to the combined effects of enhanced behavioral expression and increased glucose availability around farrowing. Because the amount of enrichment material consumed was not quantified, the contribution of its nutritional value remains speculative and should be interpreted with caution. Nevertheless, provision of enrichment material also increased piglet glycemia at birth. Higher neonatal glycemia has been associated with improved vitality and survival potential (Uddin et al. 2022), although no differences in piglet survival were observed in the present study. Instead, piglets born from enriched sows exhibited greater weaning weights, suggesting that the benefits of enrichment may be reflected in offspring growth performance rather than survival under the conditions of the present experiment.

Another factor that warrants consideration and remains insufficiently explored in relation to enrichment material provision is the link between maternal behavior, stress and pain perception. Farrowing is a well known painful moment for sows (Mainau and Manteca 2011; Ison et al. 2018). Stress is known to lower pain thresholds and increase sensitivity to nociceptive stimuli (Espejo Beristain et al. 2022). Elevated anxiety may stimulate catecholamine secretion. This response can enhance nociceptive signaling from the pelvic region and its cortical processing (Lowe 2002). During the peripartum period, this physiological stress may be intensified by restrictive housing and the absence of enrichment materials. Together, these factors may contribute to increased behavioral indicators of pain (Ison et al. 2018), including changes in facial expressions that have recently been recognized as reliable indicators of pain in farrowing sows, and could lead to an unwanted maternal behavior (Navarro et al. 2020).

The facial expression scale proposed by Navarro et al. (2020) assesses five facial action units that change according to pain intensity during parturition. Considering that the Navarro et al. (2020) facial expression scale ranges from 0 to 10, CONT sows exhibited facial expression scores consistent with the presence of pain-related facial changes, with values approaching 4 at the highest points. In contrast, providing abundant nesting material (BN15) reduced these pain-related facial expression scores by nearly half, suggesting a lower expression of facial features associated with pain or discomfort during farrowing. These findings should be interpreted as changes in facial features associated with pain or discomfort rather than as direct evidence of reduced pain. More broadly, recent reviews have concluded that grimace scales are a promising and reliable tool for identifying acute pain in domestic animals, including sows, although further validation against behavioral and physiological indicators is still required, particularly for quantifying pain intensity (Fischer-Tenhagen et al. 2022; Ison et al. 2018).

One possible explanation for this result is that access to nesting material reduced stress associated with the farrowing environment. Oxytocin has been proposed as a potential mediator linking environmental enrichment, stress regulation, maternal behavior, and analgesia during parturition (Rash et al. 2014; Li et al. 2020; Suzuki et al. 2023; Uvnäs-Moberg 2024). However, because oxytocin was not measured in the present study, this mechanism remains speculative. Further studies combining facial expression assessments with physiological indicators, including endocrine and neuroendocrine measurements, are needed to clarify the mechanisms linking enrichment material provision and sow responses during farrowing. To our knowledge, this study is the first to report an association between the provision of nest-building material and facial expression scores indicative of pain in sows around farrowing. These findings highlight the need for further research to better understand the relationships among nest-building behavior, environmental conditions, stress, and pain perception during parturition, and how these factors may collectively influence sow welfare.

The biopolymer-based enrichment material evaluated in this study is biodegradable and highly water-soluble, disintegrating upon contact with water and thereby reducing the risk of blockage in manure handling systems, a common limitation associated with conventional nesting materials. These characteristics allow the provision of larger quantities of enrichment material in commercial farrowing systems. The present results demonstrate that providing prepartum sows with this biodegradable and water-soluble enrichment material promoted nest-building behavior, reduced redirected nest-building behavior and facial expression scores associated with pain, increased sow and piglet glycemia, improved the pattern of piglet birth intervals, and increased piglet weaning weight. Collectively, these findings suggest that such materials may represent a practical strategy to improve behavioral expression and performance-related outcomes in farrowing-crated sows.

## Conclusion

Providing prepartum sows with a biopolymer-based enrichment material facilitated the expression of nest-building behavior during the prepartum period in crated sows and also increased sow glycemia. Consequently, behavioral indicators of stress, discomfort, and facial expression scores associated with pain were reduced, which was associated with improved farrowing outcomes, sow welfare, and enhanced piglet performance. Our findings indicate that, for crated sows, provision of at least 3 kg of this novel biopolymer-based material can serve as an effective alternative to natural materials, with the added benefit of not posing a risk of slurry system blockage. Future research should investigate the mechanisms underlying the observed reduction in pain in sows with access to enrichment materials and further evaluate the long term effects on offspring welfare and performance.

## Data Availability

The data sets and materials are available from the corresponding author on request.
